# Management of Acute Kidney Injury Following Major Abdominal Surgery: A Contemporary Review

**DOI:** 10.3390/jcm9082679

**Published:** 2020-08-18

**Authors:** Joana Gameiro, José Agapito Fonseca, Filipe Marques, José António Lopes

**Affiliations:** Division of Nephrology and Renal Transplantation, Department of Medicine, Centro Hospitalar Lisboa Norte, EPE. Av. Prof. Egas Moniz, 1649-035 Lisboa, Portugal; jose.nuno.agapito@gmail.com (J.A.F.); filipedcmarques@campus.ul.pt (F.M.); jalopes93@hotmail.com (J.A.L.)

**Keywords:** acute kidney injury, abdominal surgery, diagnosis, treatment

## Abstract

Acute kidney injury (AKI) is a frequent occurrence following major abdominal surgery and is independently associated with both in-hospital and long-term mortality, as well as with a higher risk of progressing to chronic kidney disease (CKD) and cardiovascular events. Postoperative AKI can account for up to 40% of in-hospital AKI cases. Given the differences in patient characteristics and the pathophysiology of postoperative AKI, it is inappropriate to assume that the management after noncardiac and nonvascular surgery are the same as those after cardiac and vascular surgery. This article provides a comprehensive review on the available evidence on the management of postoperative AKI in the setting of major abdominal surgery.

## 1. Introduction

Acute kidney injury (AKI) is defined by a rapid decrease in renal function, characterized by an increase in serum creatinine (SCr) or a decrease in urine output (UO) [1,2]. AKI is frequent in hospitalized patients and has a significant impact on patient outcomes, namely increasing in-hospital mortality, increasing length of hospital stay, increasing health care costs, contributing to the progression to chronic kidney disease (CKD), and increasing cardiovascular events [3,4,5,6,7,8,9].

The incidence of AKI has increased in past decades, reflecting the increased recognition of this syndrome, the population ageing and the burden of comorbidities, including CKD, and the increased exposure to nephrotoxins [8,10,11,12,13]. Despite a global decrease in mortality rates in recent years, it is well recognized that mortality increases with the severity of AKI and can reach up to 60% in critically ill patients [14,15,16].

Surgery is a leading cause of AKI in hospitalized patients, which can account for up to 40% of in-hospital AKI cases [17]. The highest rates are found after cardiac surgery, followed by general and thoracic surgeries [18,19]. In patients undergoing major abdominal surgery, the incidence of AKI can reach up to 35% [19,20].

Major abdominal surgery is defined as intraperitoneal approach performed under general anesthesia [21]. The pathogenesis of postoperative AKI after major abdominal surgery is complex and distinct from cardiac or vascular surgery, and includes the effects of fluid depletion, the neuroendocrine response to anesthesia and surgery itself, damage-associated molecular pattern (DAMP)-induced inflammation, as well as the role of urinary obstruction and intra-abdominal pressure [22]. Given the differences in the population and pathophysiology of postoperative AKI, it is inappropriate to assume that the risk factors for AKI after noncardiac and nonvascular surgery are the same as those after cardiac and vascular surgery.

Considering the impact of postoperative AKI and the distinct pathophysiology of AKI after major abdominal surgery, it is important to review the available evidence on AKI management in this setting. The authors provide a comprehensive and contemporary review on the management of AKI after major abdominal surgery.

## 2. Materials and Methods

We conducted the following literature searches in June 2020 through the PubMed search engine with the MeSH terms: (1) acute kidney injury, major abdominal surgery, management; (2) acute kidney injury, major abdominal surgery, treatment, renal replacement therapy; (3) acute kidney injury, major abdominal surgery, prevention.

We included articles published in English after the year 2010 up to 30 June 2020, of adult patients submitted to major abdominal surgery, evaluating the characteristics of the diagnosis and treatment of AKI in these patients and the importance of prevention of this disease.

## 3. AKI Diagnosis

Over the last decade, the definition of AKI has evolved from the former term acute renal failure to the currently used Kidney Disease: Improving Global Outcomes (KDIGO) classification which combines small changes in SCr and UO [2]. Accordingly, AKI is defined as an increase in SCr by ≥0.3 mg/dL (≥26.5 μmol/L) within 48 h, or an increase in SCr to ≥1.5 times the baseline value, which is known or presumed to have occurred within the prior seven days, or UO of less than 0.5 mL/kg/h for 6 h [2]. This classification also stratifies patients in three stages of AKI severity [2]

Recent studies demonstrate a highly variable incidence of AKI following major abdominal surgery, with the majority of patients in all studies classified as lesser stages of AKI severity (Risk or Stage 1), however, only a minority of these studies simultaneously assessed SCr and UO, as recommended, which could be a significant reason for the heterogeneity of the reported incidence [19,23].

Both criteria used to define AKI, namely SCr and UO, have important limitations to be noted [9,24]. The rise in SCr is often delayed 48–72 h after kidney injury, which limits the early recognition of AKI [25,26]. Moreover, SCr is influenced by several factors affecting its production (age, gender, diet, muscle mass, sepsis), dilution (fluid administration), elimination (previous renal dysfunction), and secretion (medications) which may lead to changes in SCr without real injury to the kidney [25,26]. Additionally, when renal injury occurs in the setting of appropriate renal reserve, meaning that other nephrons increase function to compensate injured nephrons, SCr may not change despite actual structural damage [25,26]. Accordingly, in the surgical setting, muscle wasting and positive fluid balance are associated with lower SCr and might lead to the underestimation of AKI.

UO is an early marker for AKI, independent of SCr, but highly dependent on patient’s volemic and hemodynamic status. It is influenced by the use of diuretics, difficult to assess without a urinary catheter, and its usefulness relies on an hourly assessment [26,27,28]. Surgical patients are often hemodynamically unstable, hypovolemic, and present with low cardiac output and elevated hormone and catecholamine levels, all of which combined with the effects of general anesthesia can induce a physiological reduction in UO which might not correlate to renal injury [29,30,31]. Thus, the physiological response to surgery by reducing UO limits its use as a reliable marker of postoperative AKI.

Moreover, SCr and UO do not account for the duration or cause of AKI [27]. Thus, the definition based on these markers might fail to identify individuals with structural kidney injury (subclinical AKI) which has also been associated with poor outcomes, and it might incorrectly identify individuals without structural kidney injury (hemodynamic AKI) [27,32].

Recent research on serum and urine biomarkers to detect subclinical AKI has identified plasma and urine neutrophil gelatinase-associated lipocalin (NGAL), urine IL-18 and albuminuria as the most promising postoperative markers mainly in cardiac procedures [33]. Indeed, in the Translational Research Investigating Biomarker Endpoints in AKI (TRIBE AKI) study, the increase of these biomarkers, at the time of creatinine-based AKI diagnosis, was associated with an increased risk of AKI progression (urine IL-18 OR 3.0 (95% CI 1.3–7.3), albuminuria OR 3.4 (95% CI 1.3–9.1), and plasma NGAL OR 7.7 (95% CI 2.6–22.5)). Additionally, these biomarkers enhanced risk assessment when compared to SCr [34]. The combination of markers has been reported to improve sensitivity of early recognition of postoperative AKI [35]. The most recent AKI markers are tissue inhibitor of metalloproteinases-2 (TIMP-2) and insulin-like growth factor binding protein 7 (IGFBP7) which have been validated in the setting of cardiac and non-cardiac surgery [36,37]. Higher median values of [TIMP-2] × [IGFBP7] immediately after surgery were associated with an increased risk of AKI within 48 h after surgery (area under the curve (AUC) 0.85 (95% CI 0.78–0.93) [36]. Measuring those biomarkers in the early phase might promptly predict an AKI event. Other promising biomarkers have also been studied, namely kidney injury molecule 1 (KIM-1), interleukin 18 (IL-18), liver-type fatty acid-binding protein (L-FABP), N-acetyl-glucosaminidase (NAG), monocyte chemoattractant protein 1 (MCP-1), calprotectin, urine angiotensinogen (AGT), and urine vanin-1, urine microRNAs [38,39,40,41,42,43,44]. Despite promising results, these novel biomarkers are not routinely used in clinical practice.

## 4. Management

The management of postoperative AKI must begin before surgery by recognizing high-risk patients and optimizing their clinical condition before and during surgery to prevent AKI occurrence. Anesthetic care during surgery is crucial to ensuring adequate organ perfusion and oxygenation during surgery. The use of surgical approaches that are less damaging for the kidneys is also important to prevent AKI, as well as postoperative monitoring of hemodynamic status and of development of surgical complications, namely postoperative infections [22].

**Figure 1:**
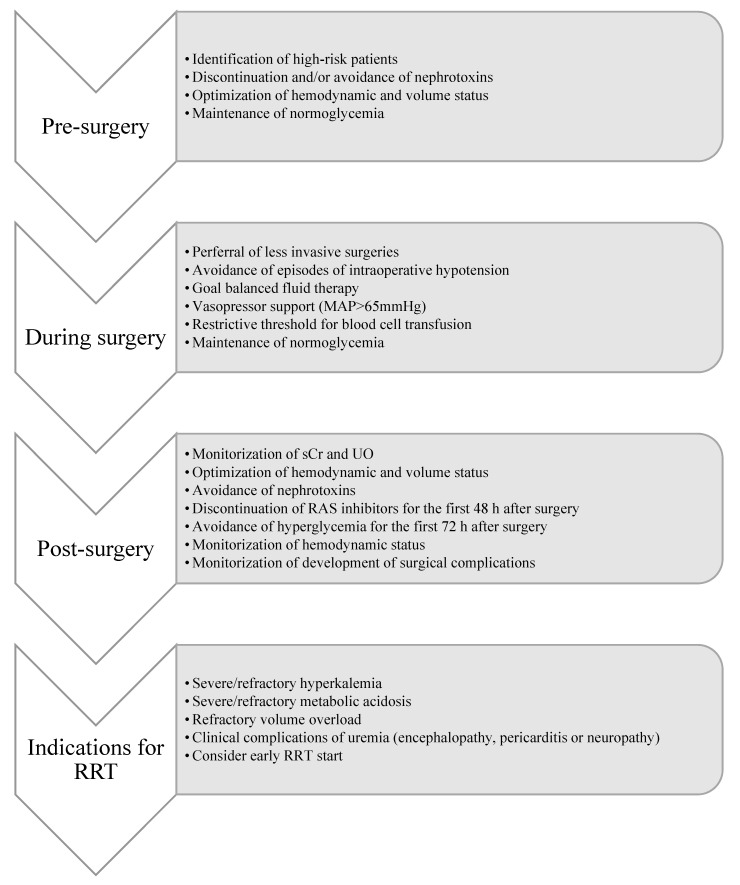
Management of AKI. MAP—mean arterial pressure; RRT—renal replacement therapy; SCr—serum creatinine; UO—urine output.

Once AKI is established, complications such as volume overload, hyperkalemia, metabolic acidosis, encephalopathy or pericarditis must be promptly treated [45]. Nephrotoxins should be discontinued or switched to other less nephrotoxic medications, and medication must be adjusted to renal function in order to prevent underdosing and adverse effects [45,46] (Figure 1).

### 4.1. Recognition of High-Risk Patients

Several patient and procedure-related risk factors have been linked with the development of AKI after abdominal surgery, namely older age, male gender, hypertension, metastatic cancer, hypoalbuminemia, diabetes mellitus, CKD, use of angiotensin-converting enzyme inhibitors or angiotensin-receptor blockers, use of intravenous contrast, use of diuretics and vasopressors, more invasive surgeries, episodes of intraoperative hemodynamic instability, need for intra-operative blood transfusions, and large colloid infusion during surgery [23,47,48]. Additionally, postoperative complications, such as leak, respiratory failure, and sepsis, are also associated with higher incidence of AKI [23,47,48] (Table 1).

Kheterpal et al. conducted a prospective observational study of 65,043 patients undergoing major noncardiac surgery and identified several preoperative and intraoperative variables as independent AKI predictors (*p* < 0.05), namely older age, emergent surgery, liver disease, body mass index, high-risk surgery, peripheral vascular occlusive disease, chronic obstructive pulmonary disease necessitating chronic bronchodilator therapy, vasopressor requirement, and diuretic requirement [49]. The use of these preoperative and intraoperative variables allowed for the creation of an AKI risk prediction model which had an acceptable AUC of 0.79 ± 0.02 [49].

Recently Park et al. developed a risk prediction model for postoperative AKI from an observational study of 51,041 patients [50]. The Simple Postoperative AKI Risk (SPARK) score included age, gender, expected surgery duration, emergency operation, diabetes mellitus, use of renin-angiotensin-aldosterone inhibitors, baseline eGFR, albuminuria, hypoalbuminemia, anemia, and hyponatremia, and performed reasonably with an AUC of 0.80 (0.79–0.81) [50].

The use of artificial intelligence algorithms and electronic alerts based on the patient health records has enhanced the development of predictive and risk stratification algorithms which have been reported to improve AKI detection [51,52,53].

Risk assessment prior to surgery is crucial to optimize strategies to prevent AKI and to determine which patients might require more intense monitorization after surgery.

### 4.2. Hemodynamic Status Management

Episodes of intraoperative hypotension, defined as mean arterial pressure (MAP) below 60 mmHg, may decrease renal perfusion, resulting in AKI in patients with impaired autoregulation [54,55,56]. Sun demonstrated that sustained intraoperative periods of MAP less than 55 mmHg (2.34 (95% CI 1.35–4.05)) and less than 60 mmHg (OR 1.84 (95% CI 1.11 to 3.06)) were associated with postoperative AKI [54]. Also, intraoperative hypotension for more than 1 min is significantly associated with an increased risk of postoperative AKI [55]. Thus avoidance of intraoperative hypotension could decrease the risk of AKI. Intraoperative blood pressure variability has also been associated with increased AKI risk due to inappropriate kidney perfusion [57].

Fluid management is complex and should be individualized to patient’s volemia [58,59]. Evidence supports the superiority of balanced crystalloids to normal saline, especially in the case of large intravenous fluid administration, due to the risk of acidosis with normal saline [60,61]. Administration of albumin appears relatively innocent, though there is no consistent evidence of survival advantage compared with crystalloids [62,63,64].

Previous fluid balance regimens administered during abdominal surgery administer 3 to 7 L of fluids on the day of surgery [65]. Current recommendations suggest avoiding gaining more than 2.5 kg [66]. Positive intraoperative fluid balance has been independently associated with increased AKI risk and increased mortality, which might be related to renal congestion and intra-abdominal pressure [67,68,69,70]. Nishimoto et al. reported that AKI was associated with increase in intraoperative fluid balance above 40 mL/kg among 5168 patients undergoing non-cardiac surgery. Furthermore, this correlation was independent of intra-operative oliguria or large amount of bleeding requiring volume resuscitation [69]. However, despite the previous belief that restrictive fluid therapy would ensure a faster recovery and better long-term outcomes after surgery, the restrictive versus liberal fluid therapy in major abdominal surgery (RELIEF) trial demonstrated at fluid restriction (restrictive group 21.5 mL/kg vs. liberal group 34.3 mL/kg) was also associated with higher incidence of postoperative AKI (8.6% vs. 5.0%, *p* < 0.001), possibly related to reduced renal perfusion [71,72]. Thus, both negative and positive fluid balance are strong risk factors for AKI.

A goal-directed therapy guided by assessment of fluid responsiveness appears to be associated with better outcomes, however interventions to optimize hemodynamics are heterogeneous in setting, design, timing and technology [73,74]. Recent guidelines suggest the use of goal-directed therapy to prevent the development or worsening of postoperative AKI [75]. Still, in the optimization of cardiovascular management to improve surgical outcome (OPTIMISE) trial, the incidence of AKI was similar between cardiac output-guided fluid associated with vasopressor treatment algorithm and usual care (6% vs. 8%, *p* = 0.80) [76,77].

After volume resuscitation, vasopressor support should be considered to avoid positive fluid balance, ensure hemodynamic stability and maintain adequate renal perfusion [78]. In surgical patients the median blood pressure target should be higher than 65 mmHg [2,79].

Anemia plays a key role in kidney hypoxia, however, transfusions have also been associated with kidney injury in the surgical setting [80,81]. Although the mechanisms are not completely understood, this might be related to reduced oxygen delivery in critical patients and red blood cell unit storage lesions in massively transfused patients [80,81]. In cardiac surgery patients, several studies suggest that a restrictive transfusion threshold of 7.5 g/dL compared to a liberal transfusion threshold of 9.5 g/dL, results in fewer red blood cell transfusions without increasing AKI risk [82,83]. Transfusion of red blood cells must also be individualized to patients’ clinical status, and targets according to patient risk and surgical specialty should be defined [59].

### 4.3. Pharmacological Interventions

Currently, there is no evidence to support any pharmacological interventions to prevent or treat AKI, namely the use of continuous infusions of dopamine or its analogues, diuretics, use of angiotensin-converting enzyme inhibitors (ACEIs) or angiotensin II receptor blockers (ARBs) ACE inhibitors, N-acetyl cysteine (NAC), atrial natriuretic peptide (ANP), statins, dexmedetomidine, sodium bicarbonate, erythropoietin (EPO) [84].

There has been no association between dopamine use in AKI and improvement in survival or renal function [85,86,87]. There is also no evidence concerning the use of fenoldopam in preventing AKI or improving outcomes [88,89].

Furosemide has not been associated with clinical benefits namely in preventing AKI, decreasing need for renal replacement therapy (RRT) (RR 0.99, 95% CI 0.80–1.22), renal recovery or decreasing in-hospital mortality (RR 1.11, 95% CI 0.92–1.33) [90,91,92]. Indeed, many studies have associated the use of loop diuretics with increased risk of mortality, which might be related to the delay in appropriate RRT start [92,93,94]. The use of diuretics is only recommended to manage fluid overload and electrolyte disturbances in AKI [2,94].

The use of preoperative ACEIs or ARBs has been associated with an increased postoperative AKI risk, which led to the discontinuation of these medicines as a preventive measure [95]. Nevertheless, a recent meta-analysis reported a significant association between preoperative ACEI or ARB treatment and lower incidence of AKI following cardiac and abdominal surgery (RR 0.92, 95% CI 0.85–0.99), though the effect size was not clinically significant [96].

NAC has been hypothesized to act as a systemic antioxidant, which could reduce the incidence of contrast-induced nephropathy [97]. However, the results of the recent PRESERVE trial, a randomized controlled trial of 5177 patients with chronic kidney disease who underwent angiography, do not demonstrate benefits of oral NAC versus placebo in preventing AKI following contrast exposure (OR 1.06, 95% CI 0.87–1.28, *p* = 0.58) [98]. Preoperative infusion of NAC has produced conflicting results in several studies, the majority in cardiac surgery patients, and is not recommended [99].

Sodium bicarbonate was also postulated to be renoprotective through urinary alkalization, with inconsistent results [97]. The PRESERVE trial also demonstrated no benefit in bicarbonate infusion versus normal saline in contrast induced-AKI prevention (OR 1.16, 95% CI 0.96–1.41, *p* = 0.13) [98]. The same results have been replicated the surgical setting, thus it is not recommended [100,101].

The use of statins has been associated with a reduction in the incidence of AKI due to their pleiotropic effects. In a retrospective cohort of 213347 patients who underwent major surgery statin use was associated with 16% lower probability of AKI (OR 0.84, 95% CI 0.79–0.90) [102]. Nevertheless, data remain too scarce to provide a formal recommendation.

Dexmedetomidine is a highly selective α-2 agonist used in perioperative setting due to its sedative, analgesic and anxiolytic effects, which is also associated with improved hemodynamic stability [103]. In several studies, the use of dexmedetomidine was associated with a significant reduction in AKI in cardiac surgery patients (OR 0.65, 95% CI 0.45–0.92, *p* = 0.02), although no studies have been conducted in major abdominal surgery patients [103].

The effects of ANP in AKI prevention were thought to derive from vasodilatation, inhibition of the angiotensin axis, and prostaglandin release and were only studied in cardiovascular surgery with a disappointing lack of positive results [104,105].

The renoprotective and anti-inflammatory properties of EPO are controversial. The perioperative use of EPO in the prevention and treatment of postoperative AKI has only been studied in cardiovascular surgery with conflicting results [106,107,108]. The impact of ANP and EPO on AKI prevention in major abdominal surgery patients has not been studied.

The beneficial effect of remote ischemic preconditioning (RIPC) has been increasingly studied in cardiovascular surgery. RIPC is a technique which induces multiple short cycles of ischemia and reperfusion by cuff inflation in the upper limbs, thus protect the kidneys from ischemia reperfusion injury [109]. Despite promising results in several clinical trials in preventing postoperative AKI, the use of propofol during anesthesia has been associated with a decrease in RIPC’s beneficial effect, thus further studies are required to validate this technique for clinical practice, namely in major abdominal surgery [110,111,112,113,114,115].

### 4.4. Renal Replacement Therapy

In the case of severe AKI, renal replacement therapy (RRT) might be necessary to maintain volume, electrolyte, acid-base, and uremic solute homeostasis [116]. The use of RRT in critically ill patients has been increasing over time [117]. It is estimated that 10–15% of critically ill patients require RRT, which is associated with increased mortality rates and longer ICU and hospital stays [16,118,119,120].

In critically ill patients, the most frequently used modality of RRT is continuous RRT (CRRT), despite the absence of clinical evidence of superiority over intermittent RRT in terms of survival or renal recovery [121]. CRRT is a slow continuous extracorporeal blood purification which aims to correct fluid and solute imbalance applied for 24 h or longer [122].

Over the past decades, the optimal timing of initiating RRT has been broadly debated. Also, there is no consensual definition of timing to start RRT. Bagshaw et al. conducted an observational study of 1238 critically ill patients, of which 50.5% were surgical patients, and reported that outcomes related to timing of RRT start were influenced by the definition of timing. Late RRT start defined by SCr was associated with lower mortality. However, late RRT start defined by a temporal delay after ICU admission was associated with increased mortality [123]. Timing to start RRT should be standardized and described according to the AKI classification staging, to the trends of biochemical changes or illness trajectory and to severity scores [124,125].

The conventional criteria for RRT initiation in AKI are anuria, severe/refractory hyperkalemia, severe/refractory metabolic acidosis, refractory volume overload, severe azotemia or clinical complications of uremia such as encephalopathy, pericarditis or neuropathy [116,124]. Indeed, the KDIGO guidelines recommend an emergent RRT start in the setting of life-threatening changes in fluid, electrolyte, and acid-base balance, and mention that the clinical context and trends of laboratory values be considered when deciding RRT start [2].

The purpose of an early initiation of RRT includes the prevention of severe electrolyte and acid–base imbalances, prevention of uremic complications, better management of fluid status and prevention of volume overload, prevention of unnecessary or excessive diuretic exposure (minimizing their adverse effects) and the capability of immunomodulation and clearance of inflammatory mediators which might minimize distant organ injury [116,125,126,127]. However, early initiation of RRT might expose patients who may not need RRT to several risks, such as, complications associated with vascular access placement, catheter-related bloodstream infections, exposure of blood to an extracorporeal circuit and anticoagulation, underdosing of antibiotics and other vital drugs, malnutrition, hemodynamic instability which may contribute to delayed kidney recovery, and increased health-care costs [116,125,128,129]. Thus, the theoretical benefit for earlier RRT, by avoiding AKI complications, must be weighted with the potential risk for delayed recovery or other complications related to RRT [116,124].

The evidence on the timing of RRT start in the setting of major abdominal surgery is limited to two observation studies and on randomized controlled trial combining surgical and medical patients.

Shiao et al. conducted a prospective observational study of 98 AKI patients who required RRT after undergoing major abdominal surgery. The criteria for RRT start were azotemia with uremic symptoms, oliguria or anuria, fluid overload, hyperkalemia, and metabolic acidosis, and timing was defined according to the risk, injury, failure, loss of kidney function, and end-stage kidney disease (RIFLE) classification by glomerular filtration rate (GFR) criteria. Accordingly, early dialysis group included RIFLE-0 or RIFLE-R and late dialysis group included RIFLE-I and RIFLE-F. Fifty two percent of patients were categorized as early dialysis. In this cohort, late RRT start was an independent predictor of in-hospital mortality, which supports early initiation of RRT in this setting. Interestingly, patients who started RRT due to oliguria or anuria (45.1% in the early group and 36.2% the late group) might not be considered severe by RIFLE classification as UO criteria was not used in categorizing patients [130].

The same authors also performed an observation study of 648 postoperative AKI patients, of which 28.2% underwent major abdominal surgery. Timing was categorized in three groups according to the time between intensive care unit (ICU) admission and RRT initiation, as follows, early group defined as 0 to 1-day, intermediate group defined as 2–3 days, and late group defined as more than three days. This study revealed a U- shaped curve association between the timing of RRT initiation and in-hospital mortality in postoperative AKI patients (59.0% in the early group, 47.8% in the intermediate group and 67.0% in the late group, *p* = 0.001). Nevertheless, the majority of patients who underwent major abdominal surgery patients started RRT in the intermediate and late group (19.9% in the early group, 35% in the intermediate group and 32.5% in the late group, *p* = 0.001) [131].

The early versus late initiation of RRT (ELAIN) was a single-center trial of 231 critically ill AKI patients, which included 78 patients (33.8%) who underwent abdominal surgery. The Early RRT group was defined as starting RRT within 8 h of fulfilling KDIGO stage 2 AKI, and delayed RRT was defined as starting RRT within 12 h of developing KDIGO stage 3 AKI or in the presence of an absolute indication. Only 9% of patients in the delayed group did not start RRT. In this trial early RRT was associated with 15% less mortality, greater RRT independence and less hospitalization days than the delayed RRT group [126]. Furthermore, early RRT start was associated with better long-term outcomes on a one-year follow-up, namely a decrease in renal function and dialysis dependence and a decrease in mortality [132].

The STARRT-AKI Investigators recently performed a randomized trial of 2927 critically ill patients which did not prove survival benefit in an early RRT start strategy [133]. This trial compared an accelerated RRT strategy, which was initiated within 12 h after reaching AKI KDIGO 2, and a standard RRT strategy, in which RRT was discouraged unless conventional indications developed, or AKI persisted for more than 72 h. A subgroup analysis of the 33% surgical patients which were included demonstrated that a standard RRT strategy was associated with lower mortality at 90 days 37.6% vs. 32.9% OR 1.20 (0.91–1.59). However, the surgical setting of these patients is not specified [133].

The lower SCr levels following abdominal surgery reflecting low muscle mass, increased catabolism or positive fluid balance, and the low sensitivity of oliguria as an AKI marker suggest that even inferior stages of AKI might represent a higher disease burden and RRT requirement. Despite the theoretical benefit surrounding early RRT start in surgical patients, there is no consistent evidence to contribute to a solid recommendation of the prescription of early RRT start [127,134]. Additionally, timing of treatment must not be considered alone but together with the dialysis dose applied [125].

## 5. KDIGO Bundle of Preventive Strategies

The KDIGO guidelines propose a bundle of preventive strategies for patients at high risk for the development of AKI. AKI prevention includes risk-stratification at admission, optimization of hemodynamic and volume status, discontinuation and avoidance of nephrotoxic agents such as NSAIDs, vancomycin, aminoglycosides, diuretics, and contrast media, maintenance of normoglycemia, monitoring of sCr and urine output, and functional hemodynamic monitoring [46,135]. Figure 1 summarizes the preventive and treatment strategies for AKI patients after major abdominal surgery.

The PrevAKI trial demonstrated that implementing these bundles as compared with standard care was associated with a significant decrease in AKI incidence in 1046 cardiac surgery patients (RR 16.6, 95% CI 5.5–27.9, *p* = 0.004) [46]. Recently, Göcze et al. performed a randomized trial in critically ill patients after major noncardiac surgery and reported that early implementation of KDIGO prevention bundle triggered by biomarker detection (TIMP2 × IGFBP7) significantly reduced the incidence of moderate and severe AKI (6.7% vs. 19.7%, *p* = 0.04; OR 3.43, 95% CI 1.04–11.32) [37].

Thus, the combination of novel AKI biomarkers and the KDIGO care bundle is a promising future strategy for AKI prevention and the improvement of patient outcomes.

## 6. Conclusions

The management of postoperative AKI must begin before surgery by recognizing high-risk patients and optimizing their clinical condition before surgery to prevent AKI occurrence. The management of AKI after major abdominal surgery includes hemodynamic stabilization, fluid balance control, eviction of nephrotoxins, improved preoperative patient management, and renal replacement therapy. Evidence supports an early RRT start in this setting. The KDIGO care bundle is a promising strategy for AKI prevention.

## Figures and Tables

**Table 1:** Risk factors for AKI after major abdominal surgery.

**Patient Related Factors**	
Male gender Older age Higher body mass index Chronic kidney disease Hypertension Cardiovascular disease Diabetes Chronic obstructive pulmonary disease	Metastatic cancer Hypoalbuminemia Use of angiotensin-converting enzyme inhibitors or angiotensin-receptor blockers Higher MELD, Revised Cardiac Index and SAPSII scores
**Procedure Related Factors**	
Use of intravenous contrast Use of diuretics and vasopressors Invasive procedures Intraoperative hemodynamic instability	Intra-operative blood transfusions Large colloid infusion Epidural anesthesia in liver resections
**Procedure Related Complications**	
Leak Respiratory failure Sepsis	
